# Leukemic Ischemia: A Case of Myocardial Infarction Secondary to Leukemic Cardiac Involvement

**DOI:** 10.1155/2017/7298347

**Published:** 2017-08-07

**Authors:** Dzmitry Fursevich, Colin Zuchowski, Joseph Limback, Melissa Kendall, Ashley Ramirez, Naim Fanaian, Jeremy Burt

**Affiliations:** Florida Hospital, Orlando, FL, USA

## Abstract

We report a case of a 39-year-old male who presented to the emergency department with acute chest pain while being in remission from T-cell acute lymphoblastic leukemia (T-ALL). Cardiac markers were elevated and EKG revealed ischemic changes compatible with acute myocardial ischemia. Coronary computed tomography angiography (CCTA) showed calcium-free coronary arteries and soft tissue myocardial infiltration suggestive of cardiac leukemia. A bone marrow biopsy confirmed recurrence of T-ALL, and patient was successfully treated with chemotherapy. We discuss the prospective diagnosis of myopericardial leukemic involvement and the role of CCTA in diagnosis and perform a literature review.

## 1. Introduction

In postmortem studies of lymphocytic neoplasms, cardiac involvement by leukemia and lymphoma has been reported in 8.7% to 37% cases [1]. Reports of antemortem leukemic cardiac infiltration are scarce, and in the few cases described T-cell acute lymphoblastic leukemia (T-ALL) is rarely the offender. We present a unique case of T-ALL manifesting as a non-ST-segment elevation myocardial infarction (NSTEMI), conduct a literature review, and highlight the diagnostic role of coronary computed tomography angiography (CCTA).

## 2. Case Presentation

A 39-year-old male presented to the emergency department (ED) with acute chest pain 1.5 years after initial diagnosis of T-ALL. At the time of diagnosis, the bulk of his extramedullary disease was in the chest, primarily involving the anterior and middle mediastinum. The patient was successfully treated with radiation, chemotherapy, and double umbilical cord blood transplant, with complete response to therapy. His remission status was monitored by periodic bone marrow biopsies.

**Figure 1:**
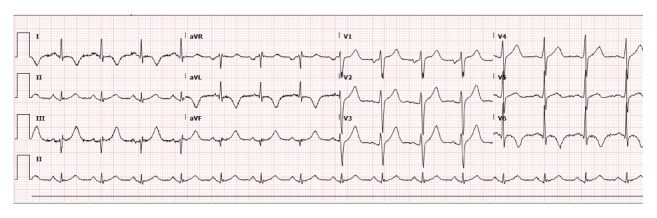
12-lead EKG manifestation of anterolateral ischemia as lateral lead T-wave inversion without ST-segment elevation.

**Figure 2:**
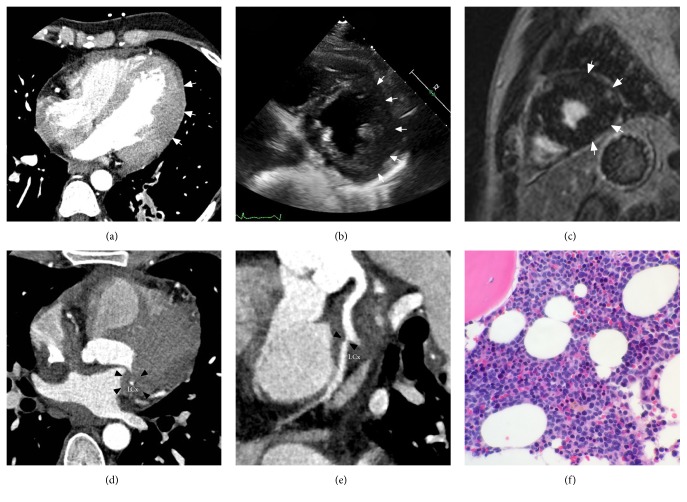
(a) Axial coronary computed tomography angiogram (CCTA), (b) echocardiogram, and (c) CMR with delayed myocardial enhancement images demonstrate marked asymmetric thickening of the lateral left ventricular wall* (arrows)*. There is obliteration of left ventricular pericardial fat on CCTA. (d) Axial and (e) curviplanar reformatted CT images of the left circumflex artery* (LCx)* show abnormal soft tissue in the left atrioventricular groove* (arrowheads)* resulting in approximately 50% stenosis of the LCx immediately after the takeoff of the first obtuse marginal branch. (f) Bone marrow biopsy slide demonstrates replacement of normal hematopoietic elements by small lymphoblasts comprising approximately 50% of total cellularity, compatible with leukemia recurrence.

At the time of ED presentation, the patient complained of acute onset dull chest pain. His troponin T was elevated at 0.2 ng/ml (normal <0.03 ng/ml). EKG showed lateral lead T-wave inversion without ST-segment elevation (Figure 1), consistent with a NSTEMI. A CCTA showed calcium-free coronary arteries; however, there was extensive soft tissue infiltration of the right atrium, atrioventricular (AV) groove, lateral myopericardium, and lateral pericardium (Figure 2). The AV groove infiltration resulted in approximately 50% narrowing of the proximal left circumflex artery. Echocardiogram and cardiac magnetic resonance imaging (CMR) also demonstrated lateral left ventricular wall thickening, and leukemia recurrence was suspected. Bone marrow biopsy confirmed T-ALL recurrence.

**Figure 3:**
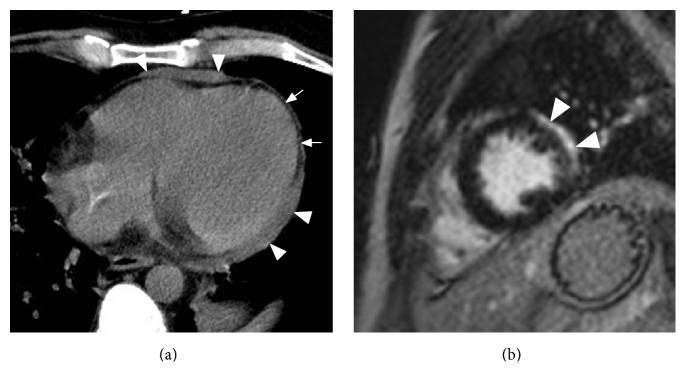
(a) Follow-up noncontrast CT after chemotherapy demonstrates marked treatment response with visualization of the pericardial fat* (arrows)* and mild residual soft tissue along the anterior RV and lateral LV* (arrowheads)*. (b) CMR DME performed for further characterization shows avid delayed enhancement of the soft tissue along the lateral LV* (arrowheads)*, compatible with posttreatment scar.

The patient was treated for NSTEMI with beta-blockade and aspirin and was immediately given methotrexate, vincristine, pegylated asparaginase, and dexamethasone chemotherapy regimen. A follow-up chest CT 5 months later demonstrated marked decrease in soft tissue cardiac and pericardial infiltrate, and repeat CMR showed focal scarring along the lateral left ventricular wall (Figure 3). Bone marrow biopsy confirmed complete response to therapy.

## 3. Discussion

Our report illustrates a unique case of T-ALL recurrence which presented with chest pain, EKG changes, and elevated cardiac enzymes. Additionally, it is remarkable that leukemia recurrence was suggested prospectively by the CCTA findings of LCx narrowing and soft tissue infiltration of the lateral left ventricular wall. Only a few reports of antemortem diagnosis of cardiac T-ALL have been discussed in the literature. For example, Prenner et al. reported a case of T-ALL initially presenting with fatigue and dry cough, with echocardiogram and CMR findings of anterolateral left ventricular involvement and decreased left systolic function [2]. De Lazzari et al. described a case of large granular lymphocyte leukemia presenting with anasarca and left ventricular dilatation, inferolateral myocardial involvement, and severe systolic dysfunction [3]. Barbaric et al. reported a case of B-cell ALL with a large leukemic right ventricular mass incidentally detected on routine pretreatment echocardiogram [4]. None of these cases utilized CCTA as the initial imaging modality of choice, and the former two required endomyocardial biopsy for diagnosis.

In our case, the diagnosis of leukemia was suggested prospectively based on the CCTA images. While there was no imaging evidence of infarction, we speculate that patient's symptoms, EKG changes, and elevated cardiac enzymes were caused by either (a) LCx narrowing, (b) microvascular obstruction from direct leukemic myocardial invasion, (c) focal pericardial invasion resulting in focal pericarditis, or (d) a combination of the previous factors. More importantly, CCTA was able to detect all of these abnormalities and suggest the diagnosis of T-ALL recurrence prospectively. It also suggested that 50% stenosis of LCx was related to extrinsic infiltration of the vessel rather than intraluminal plaque, obviating the need for cardiac catheterization and guiding immediate diagnostic workup and therapy for disease recurrence.

CCTA has become the initial modality of choice in many chest pain units [5], mostly used for evaluation of suspected coronary artery disease (CAD). It has continuously demonstrated excellent performance in CAD detection: meta-analyses of several studies utilizing 64-slice CT scanners reported sensitivities between 85 and 99% and specificities between 86 and 96% [6–9]. A prospective multicenter trial comparing the 64-slice CCTA with myocardial perfusion imaging (MPI) concluded that CCTA is superior to MPI in detection of >50% coronary artery stenosis [10]. The detection of noncoronary findings on CCTA has also been well recognized, with meta-analysis of 13 studies of a total of 11,703 patients reporting overall prevalence of extracardiac findings as high as 41% [11, 12]. Besides highlighting a unique case of chest pain and elevated cardiac enzymes which were ultimately treated with chemotherapy regimen, our report illustrates yet another reason to consider CCTA in lieu of other diagnostic techniques for chest pain workup in the emergency setting.
